# The BAND Test: Improving Reliability and Balance Testing for People with Neurocognitive Impairments: A Pilot Study

**DOI:** 10.1093/geroni/igaa057.3308

**Published:** 2020-12-16

**Authors:** Evelyn Hahn, Melissa Moore, Lindsay Neirman, Stephanie Arcadia, Stacy-Jo Krasa, Tabassum Majid

**Affiliations:** 1 Functional Pathways, Sykesville, United States; 2 Sykesville, Maryland, United States; 3 University of Maryland, Baltimore County, Baltimore, United States

## Abstract

People with neurocognitive impairments have a higher risk of falls compared to other older adults and require specific cues for evaluation. Additional options for balance testing is necessary to improve reliability and assessment of fall risk. This study established the efficacy of the novel Balance Assessment for Neurocognitive Deficits (BAND) in order to improve measurement of fall risk for people with neurocognitive impairments. The BAND was analyzed for construct validity and reliability through comparison with the Berg Balance Scale (BBS). Older adults with neurocognitive impairments (n=15) in subacute and long-term settings performed BAND and BBS assessments during therapy. Clinicians determined ambulation assistance, fall risk, and time. Calculation of intraclass correlation coefficients (ICCs), standard error of measurement (SEM), and minimal detectable change (MDC95) values was completed. Corresponding ICC values were 0.985 (95% confidence interval (95% CI), 0.956-0.995) for test-retest reliability and 0.995 (95% CI, 0.985-0.998) for inter-rater reliability. Other values included SEM=0.79 and MDC95=2.18. A linear-regression graph including Pearson’s coefficient (r) demonstrated validity through comparing BAND and BBS and showed a strong correlation (r=0.94, 95% CI, 0.825-0.98). A Bland-Altman plot was created to assess agreement between clinicians, and the mean difference was 0.2667 with 95% limits of agreement (-0.897 to 1.430). The BAND demonstrated excellent reliability and agreement for clinicians providing the test. Further research is necessary to compare the BAND with additional assessments and to demonstrate the utility in expanded populations including the community.

